# Honorary Authorship: Is There Any Chance to Stop It? Analysis of the Literature and a Personal Opinion

**DOI:** 10.3390/tomography7040067

**Published:** 2021-11-15

**Authors:** Emilio Quaia, Filippo Crimi’

**Affiliations:** Department of Medicine-DIMED, Radiology Institute, University of Padova, 35100 Padova, Italy; filippo.crimi@unipd.it

Honorary authorship corresponds to the intentional misrepresentation of credit to an individual whose contributions to a biomedical article do not meet the criteria for authorship established by the International Committee of Medical Journal Editors (ICMJE) [1]. The most frequent example of honorary authorship refers to those who are named as authors —despite having had little to do with the work involved in publishing original research reports—merely because they hold senior positions within the department where the research occurred and may have helped secure funding or simple supervision. 

Honorary authorship is actually a form of guest authorship, which may manifest in three forms. Guest authorship refers to senior authors who are included because of their respect or influence in the hope that this will increase the likelihood of publication and/or impact of the paper once published [2]. Gift authorship refers to the practice of offering authorship to a senior or junior colleague in the hope that they will return the favor [2]. Finally, honorary authorship refers to those who are named as authors merely because they hold senior positions within the service or facility where the research occurred and may have helped secure funding [2]. 

Most journals now request authors to declare each individual contribution in the final article. The ICMJE [1] recommends that authorship should be based on the following four criteria, and all authors are required to individually attest to fulfill each of them: (i) substantial contributions to the conception or design of the work, or the acquisition, analysis, or interpretation of data for the work; (ii) drafting of the work or revising it critically for important intellectual content; (iii) final approval of the version to be published; (iv) agreement to be accountable for all aspects of the work in ensuring that questions related to the accuracy or integrity of any part of the work are appropriately investigated and resolved. All individuals who meet the first criterion should have the opportunity to participate in the review, drafting, and final approval of the manuscript. These authorship criteria are intended to reserve the status of author for those individuals who deserve credit and can take responsibility for the work. All those designated as authors should meet all four criteria for authorship, and all who meet the four criteria should be identified as authors. Those who do not meet all four criteria should be only cited in the acknowledgment section of the paper. Authorship also implies responsibility and accountability for published work.

There is an increased pressure on researchers to publish since bibliometric indices including number of citations, H index, and journal impact factors are considered relevant for the progression of individual academic careers. This heavy pressure on researchers to publish and to increase their bibliometric indices encourages them to put their names to papers that they may not have contributed to in a meaningful way. 

Most people consider guest authorship to be less unethical than falsification of data, and, of course, this should not be the case. All types of guest authorship—guest, gift, or even honorary authorship—may represent opportunistic behavior and scientific misconduct, and honorary authorship actually represents the most serious and frequent scientific misconduct. Honorary authorship inflates the bibliography of the honorary author, while it limits recognition of the input of those authors who meet the criteria for authorship and distorts the promotion criteria for those aspiring to an academic career. Consequently, honorary authorship is a major ethical problem for biomedical journals, which should take immediate actions to reduce its incidence. 

Articles in major radiology research journals have periodically noted the problem of honorary authorship [3,4,5,6]. A recent study by Eisenberg et al. [7] showed a high rate (26.0%) of perceived honorary authorship among first authors of original articles. An even higher frequency (58.9%) of first authors indicated that one or more co-authors did not match all the criteria defined by the ICMJE. Reports of honorary authorship were substantially more frequent among respondents of lower academic rank and in those working in an environment in which their section or department head was automatically listed as an author. The perceived honorary authorship is substantially higher among respondents from Asia and Europe than from North America [7].

A possible solution to minimize honorary authorship could be to consider for academic promotion or grant allocations mainly those papers in which the author is in the first or second place, since, traditionally, those positions are covered by those individuals who are actually the lead authors and generally considered the major contributors to the published research. To support this proposal, some journals allow shared first authorship to indicate that two or more authors who have worked together on a publication and contributed equally. Although many journals follow guidelines set by ICMJE, no criteria have been outlined for defining first authorship, nor have any recommendations been made in regard to author order [8]. The weakness of this approach would be that the role of the traditional first author is fading in multicenter studies as multi-author collaborative research and publications grow. Conversely, a more extended option of stating more than one first author could overcome this limitation, even in multicentric studies, thereby allowing the authors who substantially led the research to be highlighted. Although ICMJE criteria would be sufficient to justify the authorships, and, in many papers, it is stated that all authors fulfilled the ICMJE criteria, in almost 60% of cases, one or more of the coauthors did not fulfill those criteria [7]. Hence, it is mandatory to find other ways in order to limit these misconducts, such as setting a predefined number of authors even for multicentric studies, thus forcing the research group to include only those who actually contributed to the preparation of the study and of the manuscript.

While acknowledging the increasing complexity and often multidisciplinary collaborative nature of high-quality research, a second possible solution could be to limit the maximal allowed author number in a paper, especially in monocentric studies. Although there is no limit to the number of authors, most journals set a maximum of 9 to 12 authors, and this approach appears to be increasing in popularity. A further possible solution could be to limit the number of authors who could declare to entirely fulfill each of the four criteria of ICMJE. Journals should reserve the right to request more details about authors’ specific individual contributions if the number of authors seems disproportionate to the work that was performed. Particular attention should be paid to those papers that do not involve original research but instead summarize opinions; provide recommendations; or interpret evidence in the form of practice guidelines, consensus papers, or position statements [1]. This type of papers should most likely include a statement at the end of the paper outlining those who endorse the recommendations of the paper and/or contributed to the paper in a minor way instead of longlists of authors, and authorship should be reserved to those who actually led the research process and manuscript preparation [1]. In order to overcome this problem, international scientific societies and working groups often request authors to establish authorship criteria at the beginning of the project, limiting the number of authors for each center and thus preventing, or at least reducing, the misconduct of honorary authorship.

Another issue that should be underlined is that different journals still use an unblinded peer-review system. This of course facilitates the research groups that are more known in the field compared to young researchers that could be forced to include senior authors to improve their chances of having their paper accepted.

Finally, journals should require all authors to disclose all money received from companies on a website accessible to all interested parties. This might discourage some guest authors from selling their name and industry from paying them [9].
